# Endovascular Treatment for Acute Ischemic Stroke With or Without General Anesthesia: A Matched Comparison

**DOI:** 10.1161/STROKEAHA.121.034934

**Published:** 2022-03-28

**Authors:** Benjamin Wagner, Johannes Lorscheider, Andrea Wiencierz, Kristine Blackham, Marios Psychogios, Daniel Bolliger, Gian Marco De Marchis, Stefan T. Engelter, Philippe Lyrer, Patrick R. Wright, Urs Fischer, Pasquale Mordasini, Stefania Nannoni, Francesco Puccinelli, Timo Kahles, Giovanni Bianco, Emmanuel Carrera, Andreas R. Luft, Carlo W. Cereda, Georg Kägi, Johannes Weber, Krassen Nedeltchev, Patrik Michel, Jan Gralla, Marcel Arnold, Leo H. Bonati

**Affiliations:** Department of Neurology (B.W., J.L., G.M.D.M., S.T.E., P.L., L.H.B.), University Hospital Basel and University of Basel, Switzerland.; Clinical Trial Unit (A.W., P.R.W.), University Hospital Basel and University of Basel, Switzerland.; Institute of Diagnostic and Interventional Neuroradiology (K.B., M.P.), University Hospital Basel and University of Basel, Switzerland.; Department of Anesthesiology (D.B.), University Hospital Basel and University of Basel, Switzerland.; Neurology and Neurorehabilitation, University Department of Geriatic Medicine FELIX PLATTER and Department of Clinical Research, University of Basel, Switzerland (S.T.E.).; Department of Neurology (U.F., M.A.), Inselspital, Bern University Hospital, University of Bern, Switzerland.; Institute of Diagnostic and Interventional Neuroradiology (P.M., J.G.), Inselspital, Bern University Hospital, University of Bern, Switzerland.; Department of Neurology, Lausanne University Hospital, Switzerland (S.N., F.P., P.M.).; Department of Neurology, Cantonal Hospital Aarau, Switzerland (T.K., K.N.).; Stroke Center EOC, Neurocenter of Southern Switzerland, Ospedale Regionale di Lugano (G.B., C.W.C.).; Department of Neurology, University Hospital Geneva, Switzerland (E.C.).; Department of Neurology, University Hospital Zurich, Switzerland (A.R.L.).; Cereneo Center for Neurology and Rehabilitation, Vitznau, Switzerland (A.R.L.).; Department of Neurology (G.K.), Cantonal Hospital St. Gallen, Switzerland.; Institute of Diagnostic and Interventional Neuroradiology (J.W.), Cantonal Hospital St. Gallen, Switzerland.; Research Department, Reha Rheinfelden, Switzerland (L.H.B.).

**Keywords:** anesthesia, general, intracranial hemorrhage, ischemic stroke, propensity score, registries

## Abstract

Endovascular treatment in large artery occlusion stroke reduces disability. However, the impact of anesthesia type on clinical outcomes remains uncertain.

**Methods:**

We compared consecutive patients in the Swiss Stroke Registry with anterior circulation stroke receiving endovascular treatment with or without general anesthesia (GA). The primary outcome was disability on the modified Rankin Scale after 3 months, analyzed with ordered logistic regression. Secondary outcomes included dependency or death (modified Rankin Scale score *≥*3), National Institutes of Health Stroke Scale after 24 hours, symptomatic intracranial hemorrhage with *≥*4 points worsening on National Institutes of Health Stroke Scale within 7 days, and mortality. Coarsened exact matching and propensity score matching were performed to adjust for indication bias.

**Results:**

One thousand two hundred eighty-four patients (GA: n=851, non-GA: n=433) from 8 Stroke Centers were included. Patients treated with GA had higher modified Rankin Scale scores after 3 months than patients treated without GA, in the unmatched (odds ratio [OR], 1.75 [1.42–2.16]; *P*<0.001), the coarsened exact matching (n=332–524, using multiple imputations of missing values; OR, 1.60 [1.08–2.36]; *P*=0.020), and the propensity score matching analysis (n=568; OR, 1.61 [1.20–2.15]; *P*=0.001). In the coarsened exact matching analysis, there were no significant differences in National Institutes of Health Stroke Scale after 1 day (estimated coefficient 2.61 [0.59–4.64]), symptomatic intracranial hemorrhage (OR, 1.06 [0.30–3.75]), dependency or death (OR, 1.42 [0.91–2.23]), or mortality (OR, 1.65 [0.94–2.89]). In the propensity score matching analysis, National Institutes of Health Stroke Scale after 24 hours (estimated coefficient, 3.40 [1.76–5.04]), dependency or death (OR, 1.49 [1.07–2.07]), and mortality (OR, 1.65 [1.11–2.45]) were higher in the GA group, whereas symptomatic intracranial hemorrhage did not differ significantly (OR, 1.77 [0.73–4.29]).

**Conclusions:**

This large study showed worse functional outcome after endovascular treatment of anterior circulation stroke with GA than without GA in a real-world setting. This finding appears to be independent of known differences in patient characteristics between groups.

Controversy surrounds the optimal type of anesthesia during endovascular treatment (EVT) of large artery occlusion in acute ischemic stroke.^1,2^ Initially, general anesthesia (GA) with intubation was preferred by many centers to avoid patient movement. Recently, however, observational studies indicated that GA may worsen functional outcomes compared with conscious sedation without intubation or no sedation at all.^3–14^ Data from prospective randomized controlled trials (RCTs) are limited to 5 single-center studies, which have yielded inconsistent findings regarding functional outcomes depending on type of anesthesia.^15–19^ The most recent meta-analyses of RCT showed that GA might be superior.^20,21^ However, findings from centers with highly specialized anesthesia teams participating in RCTs might not be readily generalizable to a real-world setting. We, therefore, compared functional outcomes in patients receiving EVT for anterior circulation stroke with GA versus without GA in the SSR (Swiss Stroke Registry).

## Methods

This was a retrospective analysis of data from the national Swiss Stroke Registry.^22^ The registry started in January 2014 and collects a standardized dataset of all patients with acute cerebrovascular events including a follow-up assessment after 3 months, and is compulsory for all hospitals certified as Stroke Units or Stroke Centers, in line with European Stroke Organization criteria.^23^ We included consecutive patients receiving any form of EVT (intra-arterial alteplase or urokinase, any form of mechanical treatment including stent retriever, aspiration, distal retriever; with or without balloon angioplasty, permanent intracranial stenting, extracranial stenting) for acute ischemic stroke caused by large artery occlusion in the anterior circulation, at a certified Stroke Center between January 2014 and June 2017, with available data on anesthesia type and functional status on the modified Rankin Scale (mRS) after 3 months.^24^ Patients with vertebrobasilar stroke and patients with strokes occurring in-hospital were excluded. To reduce the risk of attrition bias, we defined a priori to include only data of Stroke Centers with an available 3-month follow-up mRS rate of >80%.

We compared patients treated with GA (GA group), defined by endotracheal intubation, versus those treated without GA (non-GA group), the latter including conscious sedation or no sedation at all. We had no data on conversion from initial non-GA to GA during the procedure. Such patients were assigned to the GA group in our study. The primary outcome measure was the level of functional disability on the mRS, an ordinal scale measuring the degree of neurological disability, ranging from 0—no symptoms to 6—death, assessed 3 months after EVT by a stroke neurologist during a clinical follow-up visit or if a clinical visit was not possible in a telephone interview. Secondary clinical outcomes were dependency or death at 3 months (mRS ≥3); 3-month mortality; recurrent ischemic stroke within 3 months; National Institutes of Health Stroke Scale (NIHSS)^25^ 1 day after EVT; change in NIHSS from admission to 1 day after EVT; hemorrhagic transformation and intracranial hemorrhage (ICH) on follow-up imaging, usually obtained 1 day after treatment; symptomatic ICH defined as any ICH on follow-up imaging associated with *≥*4 points worsening in NIHSS occurring within 7 days of acute ischemic stroke^26^; decompressive craniectomy. In addition, we investigated time metrics including time from hospital admission to start of EVT (door-to-groin puncture time); symptom onset to start of EVT; duration of EVT; as well as duration of hospital stay. Furthermore, we investigated the rate of transfer to intensive care unit (ICU) after treatment. The present analysis was approved by the Ethics Committee of Northwestern Switzerland for all contributing hospitals. Our analysis was conducted according to the Strengthening the Reporting of Observational Studies in Epidemiology criteria for observational studies.

### Matching and Statistical Analysis

Statistical analysis was performed by A.W. using R version 3.6.1 (R Core Team. 2019: R: A Language and Environment for Statistical Computing. R Foundation for Statistical Computing, Vienna, Austria). We compared the primary outcome between GA and non-GA (reference group) with ordered logistic regression models. Time from hospital admission to start of EVT, symptom onset to start of EVT, duration of EVT, and duration of hospital stay were analyzed with log-linear regression models. NIHSS after 1 day and change in NIHSS were analyzed with a linear regression model and all other secondary outcomes with logistic regression models.

**Figure 1.**
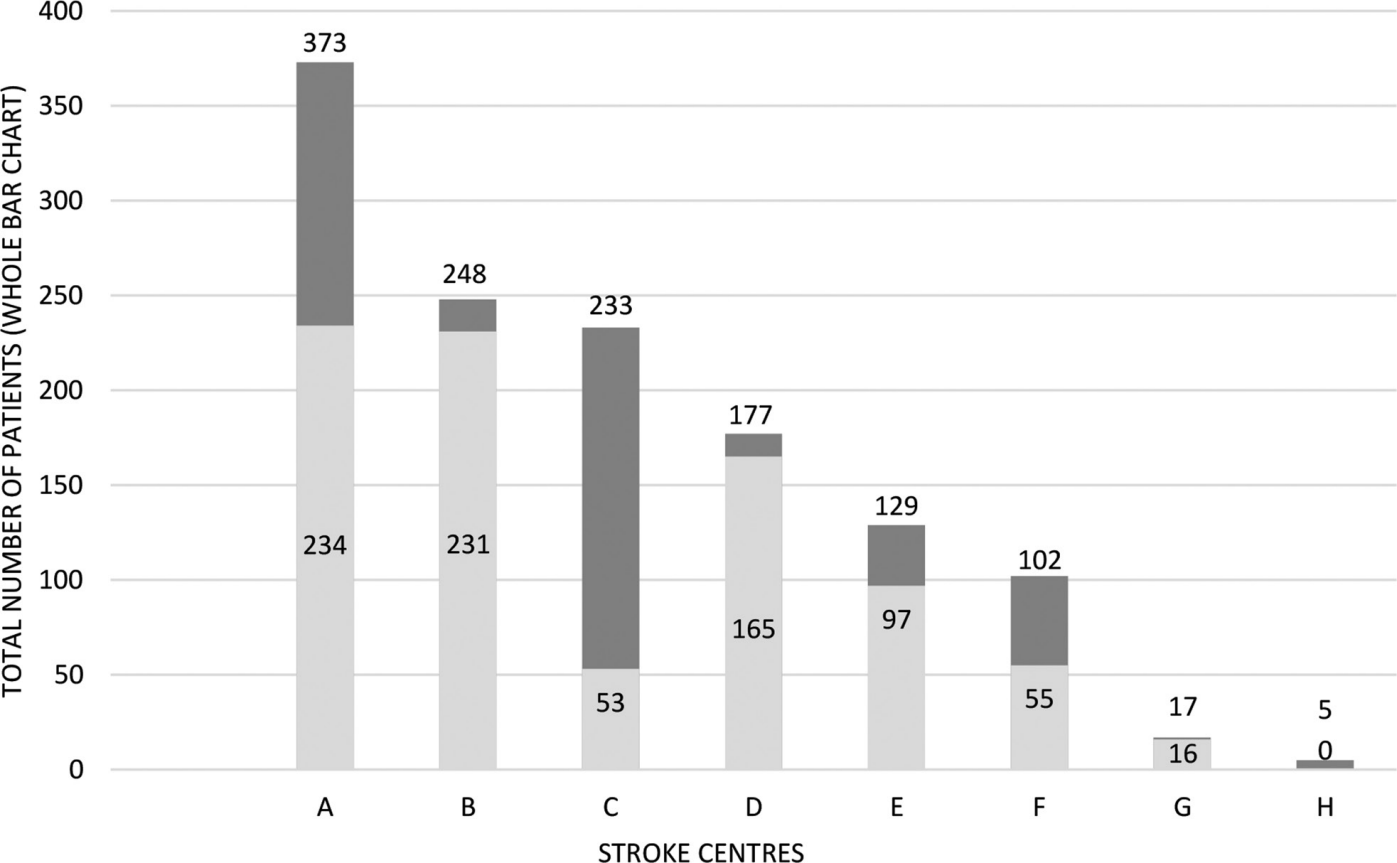
Use of anesthesia in the 8 participating Stroke Centers in Switzerland (A–H); light gray denotes patients treated with general anesthesia (GA); dark gray denotes patients treated with non-GA.

The first, unmatched analysis used the entire data set and only considered the prehospital mRS as a potentially confounding factor. Second, when estimating causal effects using observational data, it is desirable to replicate a randomized experiment as closely as possible by obtaining treated and control groups with similar covariate distributions. This goal can often be achieved by choosing well-matched samples of the original treated and control groups, thereby reducing bias due to the covariates. Therefore, we adjusted for prespecified baseline variables which might potentially confound allocation to the type of anesthesia and/or clinical outcomes using coarsened exact matching (CEM), using the R package *cem*.^27^ CEM achieves lower levels of imbalance, model dependence, and bias than propensity score matching (PSM).^28^ The basic idea of CEM is to first coarsen each variable before applying exact matching to the coarsened data. Units in strata containing at least one treated and one control unit are retained in the matched data set. To account for the different numbers of units in each stratum, the average treatment effect was obtained as a weighted estimate.^29^ The prespecified matching variables were sex, age, NIHSS at admission, involvement of single or multiple vascular territories, time from symptom onset to admission, and prehospital mRS. As the baseline variables used for matching were missing in some patients, we applied multiple imputation using the R package *Amelia* before CEM matching.^30^ We did not impute outcome data. Additionally, to account for a potential center effect we matched only patients within categories of centers with similar preferences for type of anesthesia, defined as >70% non-GA (2 Stroke Centers), >70% GA (4 Stroke Centers), or no clear preference (2 Stroke Centers) among the included patients (Figure 1).

As a sensitivity analysis, we performed PSM for cases with complete data on the abovementioned variables, using the *MatchIt* package.^31^ The propensity score was based on a multivariable logistic regression with allocation to type of anesthesia as the outcome variable and the abovementioned variables as independent variables. For PSM, we included the Stroke Center variable as a fixed effect in the propensity model. Patients were then matched in a 1:1 ratio with nearest-neighbor matching within a caliper of 0.2 SD of the propensity score without replacement. Observed differences were considered significant at *P*≤0.05.

## Results

**Figure 2.**
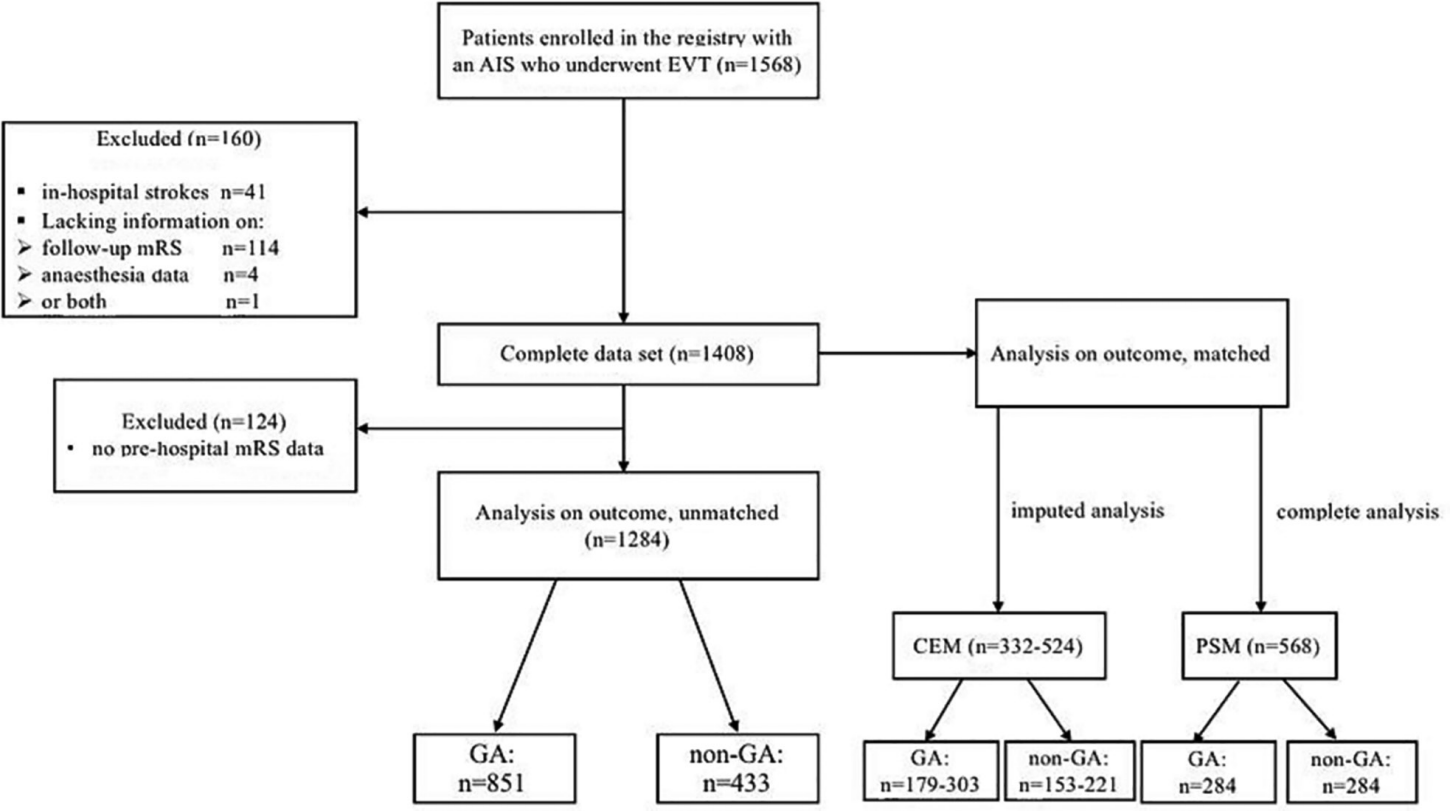
**Flow diagram of the patients included in the study.** AIS indicates, acute ischemic stroke; CEM, coarsened exact matching; EVT, endovascular treatment; GA, general anesthesia; mRS, modified Rankin Scale; and PSM, propensity score matching.

Eight of 9 Swiss Stroke Centers achieved the predefined follow-up rate. At these sites, 1568 patients with anterior circulation stroke received EVT during the inclusion period, in whom mRS at 3 months was available in 93%. Figure 2 shows reasons for exclusion of patients from the analysis. The complete data set of 1408 patients was used to build the GA and non-GA study groups with the CEM (n=269) and the PSM (n=568) matching procedure (Tables S1 and S2). For the unmatched analysis, additional 124 patients without available prestroke mRS were excluded resulting in 1284 patients, of whom 851 (66%) were treated with GA and 433 without GA (Table 1). Patients in the GA group had a higher NIHSS at admission, were older, more often had acute ischemic strokes involving multiple vascular territories, worse prehospital functional status, and a shorter symptom onset to admission time than patients without GA (Table 1). Both groups had similar prevalence of previous cerebrovascular events and vascular risk factors, with the exception of hyperlipidemia which was more common in the GA group (68.8 versus 54.8%; Table 1). Details of affected vascular territories are provided in Table S3, and endovascular treatment modality as well as rates of intravenous bridging thrombolysis in Table S4.

**Table 1.**
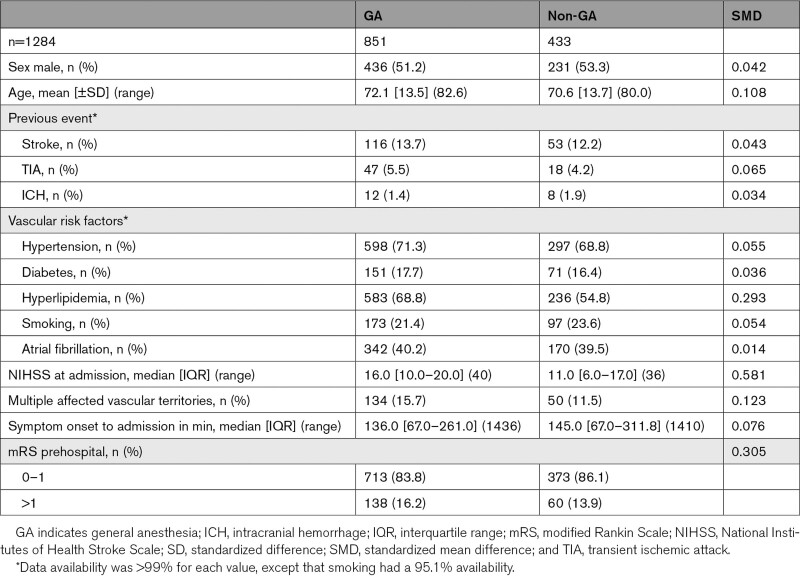
Patient Baseline Characteristics by Type of Anesthesia (Unmatched Analysis)

There was no major change in the proportion of patients treated with GA over the period of inclusion in the study, although a slight trend towards a decrease in GA use was observed (Figure S1). A single Stroke Center treating 27 patients during the inclusion period was excluded from the analysis because the site did not achieve the predefined follow-up rate.

**Figure 3.**
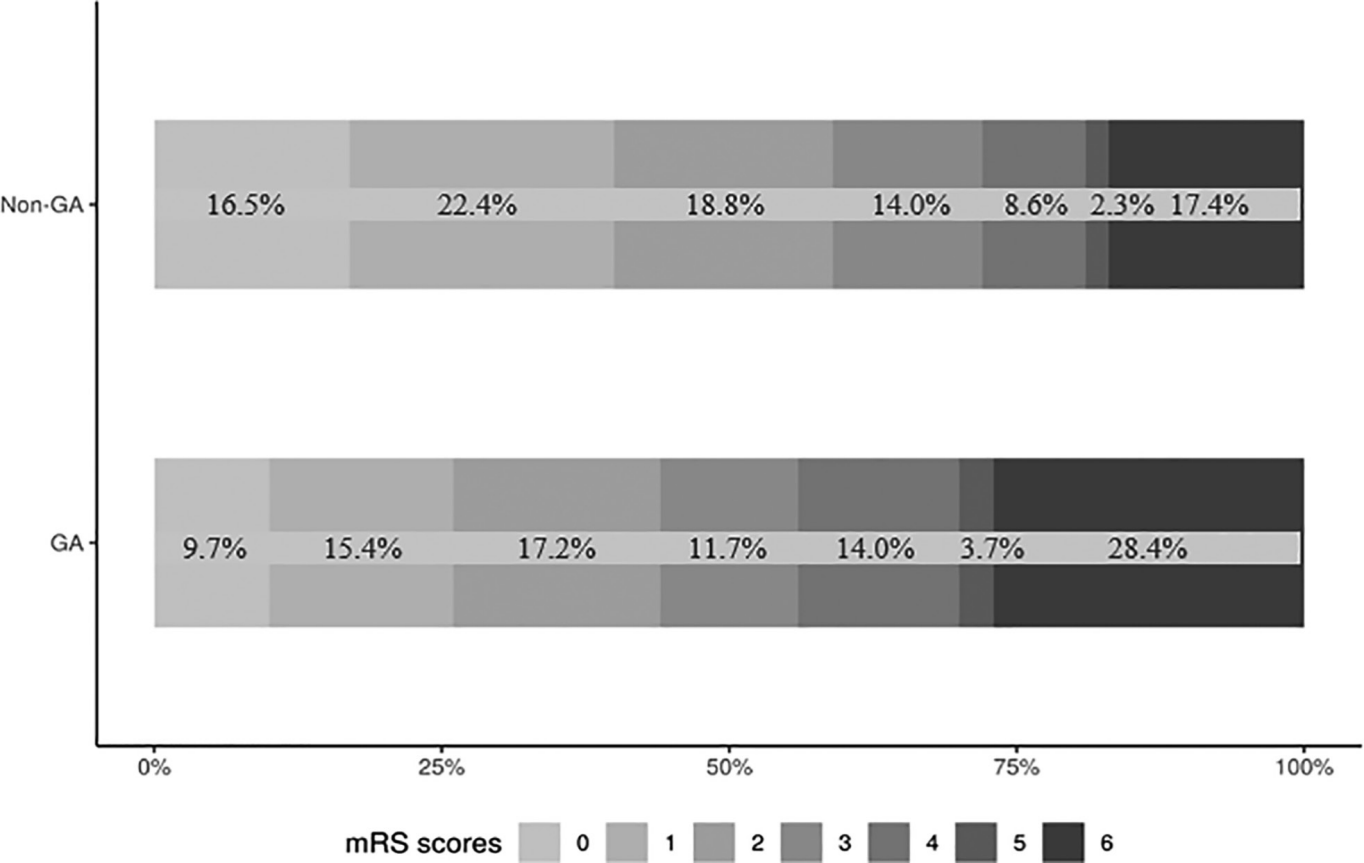
Distribution of modified Rankin Scale (mRS) 3 mo after stroke, unmatched. GA indicates general anesthesia.

In the unmatched analysis, 3 months mRS was higher in the GA group than in the non-GA group (odds ratio [OR], 1.75 [1.42–2.16]; *P*<0.001; Table 2, Figure 3). In the CEM analysis, the imputed analysis sets ranged from 332 to 524 patients, and patient characteristics were well balanced between groups with standardized mean differences <0.1 (Table 3); mRS at 3 months remained significantly higher in the GA group (OR, 1.60 [1.08–2.36]; *P*=0.020; Table 2). This was confirmed in the complete-case PSM analysis including 568 patients (OR, 1.61 [1.20–2.15]; *P*=0.001; Table 2).

**Table 2.**
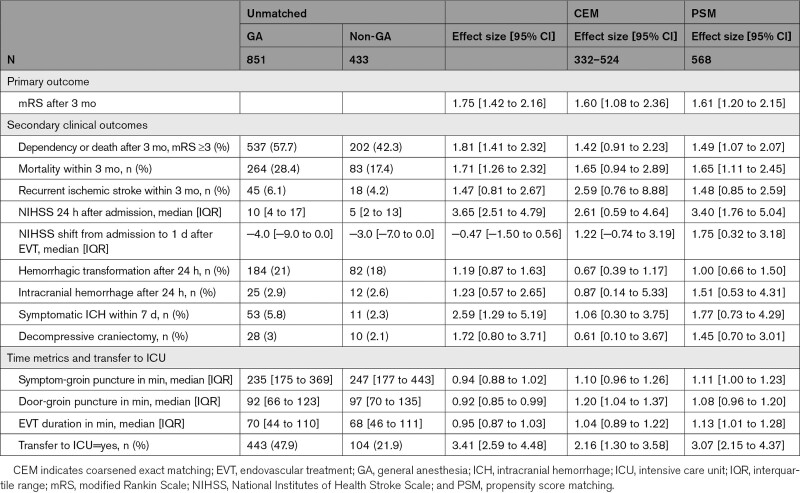
Primary and Secondary Outcomes, Unmatched, and Matched

**Table 3.**
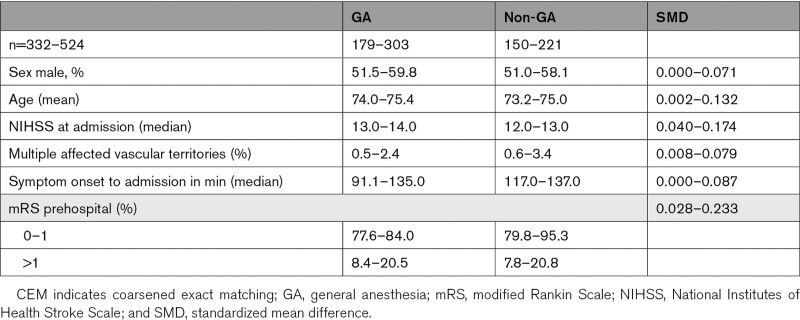
Patient Baseline Characteristics of the CEM-Matched Data Sets, as Minimum and Maximum Percentage/Mean/Median/SMD

Secondary outcomes also differed between the groups in the unmatched comparison: door-to-groin puncture time was 8% shorter in the GA group than in the non-GA group (multiplicative effect, 0.92 [0.85–0.99]). Patients in the GA group were more often transferred to ICU after treatment (OR, 3.41 [2.59–4.48]) and still had a higher NIHSS after 1 day (estimated coefficient, 3.65 [2.51–4.79]) than patients in the non-GA group; however, the change in NIHSS from baseline to 1 day did not differ between groups (4.0 [−9.0 to 0.0] versus −3.0 [−7.0 to 0.0], estimated coefficient, −0.47 [−1.50 to 0.56]). Furthermore, patients in the GA group had more often symptomatic ICH (OR, 2.59 [1.29–5.19]), 13% longer hospital stay (multiplicative effect, 1.13 [1.02−1.24]), a higher rate of dependency or death (OR, 1.81 [1.41−2.32]), and mortality (OR, 1.71 [1.26−2.32]) at 3 months than patients in the non-GA group. The other secondary outcomes, including symptom onset to start of EVT (multiplicative effect, 0.94 [0.88−1.02]), EVT duration (multiplicative effect, 0.95 [0.87−1.03]), hemorrhagic transformation (OR, 1.19 [0.87−1.63]), and ICH (OR, 1.23 [0.57−2.65]) on follow-up imaging, decompressive craniectomy (OR, 1.72 [0.80−3.71]), and recurrent ischemic stroke within 3 months (OR, 1.47 [0.81−2.67]) did not differ significantly (Table 2).

In the CEM analysis, door-to-groin puncture time was 20% longer (multiplicative effect, 1.20 [1.04−1.37]), transfer to ICU more frequent (OR, 2.16 [1.30−3.58]), and duration of hospital stay 26% longer (multiplicative effect, 1.26 [1.04−1.53]) in the GA group (Table 2). The other secondary outcomes including symptom onset to groin puncture (multiplicative effect, 1.10 [0.96−1.26]), EVT duration (multiplicative effect, 1.04 [0.89−1.22]), NIHSS after 1 day (estimated coefficient, 2.61 [0.59−4.64]), change in NIHSS (estimated coefficient, 1.22 [−0.74 to 3.19), hemorrhagic transformation (OR, 0.67 [0.39−1.17]) or ICH on follow-up imaging (OR 0.87 [0.14,5.33]), symptomatic ICH (OR, 1.06 [0.30−3.75]), decompressive craniectomy (OR, 0.61 [0.10–3.67]), recurrent ischemic stroke (OR, 2.59 [0.76−8.88]), dependency or death (OR, 1.42 [0.91−2.23]), and mortality (OR, 1.65 [0.94−2.89]) at 3 months did not differ significantly.

In the sensitivity PSM analysis, patients who received GA had 13% longer duration of EVT (multiplicative effect, 1.13 [1.01−1.28]), were more often transferred to ICU (OR, 3.07 [2.15−4.37]), had higher NIHSS at 1 day (estimated coefficient, 3.40 [1.76−5.04]), a higher rate of dependency or death (OR, 1.49 [1.07−2.07]), and mortality (OR, 1.65 [1.11−2.45]) at 3 months (Table 2).

## Discussion

We found worse functional outcomes measured by 3 months mRS in patients receiving EVT under GA compared with patients treated without GA in anterior circulation stroke at certified Swiss Stroke Centers. This result appears to be independent of known differences in characteristics of patients as it was confirmed in 2 separate analyses using methods to match patients.

Our main finding is in line with several observational studies,^3–14,32,33^ systematic reviews, and meta-analyses.^34–41^ In particular, the 2 largest single studies to date, a meta-analysis of individual patient data from 1764 patients receiving EVT in RCTs by the HERMES (Highly Effective Reperfusion Evaluated in Multiple Endovascular Stroke Trials) collaboration, and 2 registry cohort studies from Italy and Germany of EVT in acute stroke with 4429 and 5808 patients, respectively, showed a worse functional outcome after EVT under GA compared with non-GA.^32,33,36^ Recently, GA versus conscious sedation versus local anesthesia alone were compared among 1376 patients in the MR CLEAN (Multicenter Randomized Clinical Trial of Endovascular Treatment for Acute Ischemic Stroke in the Netherlands) registry yielding best functional outcome with local anesthesia.^42^

Five RCTs of limited size have compared outcomes after EVT with versus without GA: The AnStroke trial (Anesthesia During Stroke) reported no difference in 3 months mRS between GA and non-GA in 90 patients.^18^ The SIESTA trial (Sedation Versus Intubation for Endovascular Stroke Treatment) randomized 150 patients and reported no significant difference in the overall distribution of the mRS after 3 months either, but significantly more patients in the GA group were functionally independent after 3 months (mRS score of 0–2).^15^ The GOLIATH trial (General or Local Anesthesia in Intraarterial Therapy) enrolled 128 patients and reported significantly lower mRS scores at 3 months in the GA group, but no significant difference in functional independence (mRS score of 0–2).^17^ A pooled analysis of individual patient data of the RCTs SIESTA, AnStroke, and GOLIATH (n=368; GA: 183 versus non-GA: 185) demonstrated a worse functional outcome after 3 months by ordinal analysis of mRS in the non-GA groups (OR, 1.58 [1.09–2.29]).^20^ Two further smaller RCTs found no difference in 3 months mRS, and functional independence, respectively.^16,19^

Although observational studies have an inherent risk of bias, they complement the evidence provided by RCTs by measuring outcomes in less specialized settings and less selected patients than is the case in RCTs. For example, RCTs excluded patients with severe agitation^15–19^ or previous functional dependency.^16–19^ In addition, the availability of highly specialized neuroanesthesia care may have contributed to the superior outcomes with GA in the RCTs.^16,21^ Our study is among the largest to date comparing functional outcome according to the type of anesthesia in EVT in a real-world setting. We accounted for indication bias by repeating the comparison with 2 different matching approaches and were able to corroborate our main finding. Together with the evidence from previous observational studies, our data show that GA might be inferior among less selected patients and in settings with—as we assume (although details on experience of anesthesia teams and their procedures were unavailable)—potentially less specialized anesthesia teams. Our study demonstrated substantial variability in the use of GA and non-GA between the Stroke Centers in Switzerland, likely attributable to center-specific standardized operating procedures and individual physicians’ preferences. We, therefore, included Stroke Center as a matching variable in our analyses to avoid confounding of the comparison of GA versus non-GA by a center effect independent of the preference of the type of anesthesia. Arguing further against a center effect, GA tended to yield worse outcomes than non-GA at each of the 6 largest participating sites (Figure S2*).*

The main argument in favor of GA is to avoid patients’ movement and discomfort which impairs catheter navigation and interpretation of angiography.^43^ Potential disadvantages of GA include delaying the start of EVT, and a fall and/or fluctuation of arterial blood pressure.^4,5,7,9,14,44^ The SIESTA and AnStroke trial reported a 10 and 9 minutes longer time from door-to-groin puncture, but an 18 and 19 minutes shorter time from groin puncture to recanalization under GA compared with non-GA, respectively.^15,18^ In contrast, GOLIATH and CANVAS (Choice of Anesthesia for Endovascular Treatment of Acute Ischemic Stroke) showed both, a longer door-to-groin puncture time (by 9 and 14 minutes, respectively) and groin puncture to recanalization time (by 5 and 11 minutes, respectively) in the GA group compared with the non-GA group.^17,19^ In the unmatched comparison in our study, the door-to-groin puncture time was actually shorter under GA than under non-GA (92 versus 97 minutes), while the duration of EVT was similar (70 versus 68 minutes). However, these findings were not consistently replicated in the matched analyses. In addition, the relatively small differences in treatment delays observed in our study are unlikely to have had a relevant impact on 3-month outcomes.

Our findings on the remaining secondary outcomes were less conclusive. In the CEM analysis, only hospital stay duration—potentially related to the time needed for weaning and possible complications after intubation—was longer in the GA than in the non-GA group. This was also the case in the unmatched comparison but not in the PSM analysis. Unsurprisingly, the odds of being transferred to ICU after EVT were >3× higher if treatment was delivered under GA (OR, 3.41 [2.59–4.48]), indicating that extubation on the table is rarely performed after EVT. Both in the unmatched and the PSM analysis, mRS *≥*3 after 3 months, NIHSS after 24 hours, and 3 months mortality were higher in the GA than in the non-GA group, but these findings were not reproduced in the CEM analysis. Recurrent ischemic stroke within 3 months did not differ between GA and non-GA in any of the analyses. Differing outcomes in the CEM may be partly due to the fact that 124 patients with missing prehospital mRS were excluded from the other 2 analyses. Moreover, it should be noted that the secondary end points with discordant effect estimates are rather rare events resulting in a higher uncertainty of estimation, as reflected by the large width of the corresponding confidence intervals. Finally, we also cannot exclude the possibility of unobserved imbalance. Nonetheless, consistency in some of the observed differences in secondary functional outcomes such as mRS *≥*3 and NIHSS corroborate the findings of our primary analysis.

Our study has important limitations. The absence of detailed information on anesthesia management in the Swiss Stroke Registry represents a major limitation. Although studies on EVT, including ours, commonly defined GA as endotracheal intubation which required deep sedation and ventilation, various terms including “conscious sedation” or “monitored anesthesia care” are used to describe a spectrum of light to moderate sedation along with analgesia in patients treated without GA. The lack of data on respiratory parameters, arterial blood pressure, cerebral blood flow, and type of anesthetic agents used did not allow quantification of sedation or exploration of potential mechanisms underlying the association with clinical outcome. Inhaled volatile and intravenous anesthetics may cause a fall and fluctuations in arterial blood pressure.^19,45^ Hypocapnia-induced vasoconstriction of the intracranial vessels and increased cerebral venous pressure during endotracheal intubation may further reduce cerebral perfusion during GA.^44^ A fall in cerebral perfusion pressure may worsen the extent of injury to the ischemic penumbra.^6,46–49^ A systolic blood pressure below 140 mm Hg, a fall in mean arterial pressure >40% or ≥10% from baseline during EVT are considered predictors of poor neurological outcome.^3,6,50–53^ Recently, an analysis from 3 of the 5 RCTs showed an association of poor outcome in 90-day mRS scores if mean arterial pressure was below 70 mm Hg for >10 minutes or <90 mm Hg for >45 minutes.^54^ A retrospective study with 371 patients observed a linear association between the duration of arterial hypotension during EVT and the functional outcome at 3 months.^51^

We also lack data on conversion from initial non-GA to GA during the procedure, for example, due to agitation or risk of aspiration. Such patients would have been allocated to the GA group in our study. Conversion rates in the RCTs ranged from 6.3% to 18.2%^15–19^; the Italian and German Stroke Registry had conversion rates of 3.0 and 3.3%.^32,33^ Similarly, we collected no data on the timing of extubation in GA patients and whether this affected transfer to ICU. Data on pneumonia and other infections are not collected in the SSR. Whether intubation prevents pneumonia by airway protection or whether on the contrary, the risk of pneumonia is higher among ventilated patients is still a matter of debate. Three RCTs reported higher pneumonia rates in the GA group,^15,16,18^ whereas a large post hoc analysis of MR CLEAN reported a nonsignificant decrease in pneumonia under GA.^42^ Factors leading to the decision to perform EVT under GA versus non-GA in individual patients were not known. Although our study mitigated imbalances in patient characteristics by both CEM and PSM, we cannot rule out residual confounding by unmeasured factors. It should also be noted that we were only able to match 26% to 44% of the study population, which limits the generalisability of the results from the matched outcome analysis. Although both matching strategies yielded comparable results, we observed wider confidence intervals for CEM compared with PSM estimates. On one hand, the exact matching of coarsened variables in CEM leads to lower levels of covariate imbalance compared with PSM.^28^ On the other hand, CEM achieves a lower precision than PSM in settings with a relatively large number of covariates, which might explain our findings.^55^

In addition, detailed information on baseline parenchymal and vascular imaging as well as procedural success was unavailable, including the Alberta Stroke Program Early CT Score, stroke cause, the collateral circulation, the exact location of vessel occlusion (ie, arterial segment), the success of recanalization measured by Thrombolysis in Cerebral Infarction score, evidence on vessel perforation, and the final infarct volume.

Furthermore, following favorable results in clinical trials, EVT was increasingly used and evolved over the period of inclusion in our study. To address potential confounding by a temporal trend, we performed 2 post hoc analyses showing no major shift in use of anesthesia (Figure S1) and similar outcomes (Figure S3) along the years of inclusion in our study. Finally, our findings on secondary outcomes have to be interpreted with caution because of the inconsistent results in the unmatched and matched analyses.

In conclusion, in this observational study on the impact of type of anesthesia on outcome in EVT for acute ischemic stroke, we found that GA in real-world Stroke Centers was associated with worse functional outcome, which appeared to be independent of existing differences in patient characteristics between groups. Larger randomized trials are needed to study the relationship of disease-, patient- and treatment-related variables with outcomes following GA on EVT for acute ischemic stroke, and to identify the ideal type of anesthesia for individual patients.

## Article Information

### Sources of Funding

None.

### Disclosures

Dr Lorscheider reports grants from Innosuisse–Swiss Innovation Agency, grants and personal fees from Novartis, grants from Biogen, personal fees from Roche, and personal fees from TEVA outside the submitted work. Dr De Marchis reports personal fees from Bayer outside the submitted work. Dr Lyrer reports other from Bayer, Switzerland, nonfinancial support and other from Boehringer Ingelheim, other from Pfizer, grants from University Hospital Basel, Neurology Clinic, grants from Swiss National Foundation SNF, grants from ACTICOR France, other from Biogen, Switzerland, other from Alexion, and grants from Bayer, Germany outside the submitted work. Dr Fischer reports grants from Medtronic, other from Medtronic, other from Stryker, other from CSL Behring, grants from Swiss National Science Foundation, grants from Swiss Heart Foundation, and other from Boehringer Ingelheim outside the submitted work. Dr Nannoni reports grants from Swiss National Science Foundation (SNSF) outside the submitted work. Dr Luft reports personal fees from Amgen, personal fees from Moleac, and personal fees from Bayer outside the submitted work. Dr Cereda reports personal fees from iSchemaView and other from Bayer outside the submitted work. Dr Nedeltchev reports personal fees from Medtronic Switzerland, grants from Biogen Switzerland, grants from Novartis Switzerland, grants from Sanofi Switzerland, and grants from Lupin outside the submitted work. Dr Mordasini reports grants from Swiss National Science Foundation and grants from Swiss Heart Foundation during the conduct of the study. Dr Gralla reports grants from Medtronic during the conduct of the study; grants from Swiss National Funds outside the submitted work. Dr Arnold reports personal fees from Alexion, Amgen, Astra Zeneca, Bayer, Bristol Myers Squibb (BMS), Covidien, Daiichy Sankyo, Medtronic, Novartis, Pfizer, and Sanofi. Dr Bonati reports grants from Swiss Heart Foundation during the conduct of the study; grants from Swiss National Science Foundation, grants from Swiss Heart Foundation, grants from Stiftung zur Förderung der gastroenterologischen und allgemeinen klinischen Forschung sowie der medizinischen Bildauswertung, personal fees from AstraZeneca, personal fees from Claret Medical, and personal fees from InnovHeart outside the submitted work. The other authors report no conflicts.

### Supplemental Material

Tables S1–S4

Figures S1–S3

## Supplementary Material



## Appendix

### Swiss Stroke Registry Investigators

#### Stroke Center

Cantonal Hospital Aarau (KSA): Javier Anon, Sandra Clarke, Michael Diepers, Philipp Gruber, Timo Kahles, Eileen Martin, Krassen Nedeltchev, Luca Remonda, Andreas Schweikert, Vedrana Zupa. University Hospital Basel (USB): Valerian Altersberger, Kristin Blackham, Leo Bonati, Alex Brehm, Gian Marco De Marchis, Tolga Dittrich, Amgad El Mekabaty, Stefan Engelter, Joachim Fladt, Urs Fisch, Henrik Gensicke, Lisa Hert, Philippe Lyrer, Sabrina Manuzzi, Marina Maurer, Louisa Meya, Nils Peters, Alexandros Polymeris, Marios Psychogios, Sebastian Thilemann, Christopher Traenka, Ioannes Tsogkas, Benjamin Wagner, Anaelle Zietz. Inselspital, University Hospital Bern: Marcel Arnold, Urs Fischer, Martina Goeldlin, Jan Gralla, Mirjam Heldner, Simon Jung, Johannes Kaesmacher, Basel Mamaari, Thomas Meinel, Pasquale Mordasini, Madlaine Mueller, Hakan Sarykaya, David Seiffge, Bernhard Siepen, Jan Vynkier. University Hospital Geneva (HUG): Emmanuel Carrera. University Hospital Lausanne (CHUV): Ashraf Eskandari, Patrick Michel, Vasiliki Pantazou, Davide Strambo. Neurocenter of Southern Switzerland, Lugano (EOC): Giovanni Bianco, Carlo Cereda, Jane Frangi, Shairin Sihabdeen. Cantonal hospital of St. Gallen (KSSG): Georg Kägi, Jochen Vehoff. University Hospital Zuerich (USZ): Mira Katan, Andreas Luft, Achim Mueller, Susanne Wegener.
