# Supplementary material for: Endovascular Treatment for Acute Ischemic Stroke With or Without General Anesthesia: A Matched Comparison
Source: Stroke. 2022 Mar 28;53(5):1520–9. doi: 10.1161/STROKEAHA.121.034934 (PMC10082068; doi:10.1161/STROKEAHA.121.034934)
Supplement: Supplementary file 1 [file str-53-1520-s001.pdf]

Supplemental material

Supplemental tables:

Table I: Patient baseline characteristics of the complete CEM-matched data set

Table II: Patient baseline characteristics of the complete PSM-matched data set

Table III: Distribution of the affected vascular territories, unmatched

Table IV: Distribution of the endovascular treatment modality and rates of intravenous bridging thrombolysis, unmatched

Supplemental figures:

Figure I: Proportions of patients treated with and without GA from 2014 until 2017

Figure II: Distribution of modified Rankin Scale (mRS) 3 months after stroke by type of anaesthesia in the 6 largest participating centres, unmatched

Figure III: Distribution of modified Rankin Scale (mRS) 3 months after stroke by type of anaesthesia from 2014 to 2017, unmatched

<u>Supplemental tables:</u>

Table I: Patient baseline characteristics of the complete CEM-matched data set

	GA	non-GA	*SMD*
n=269	152	117	
Sex male, n (%)	85 (55.9)	71 (60.7)	*0.097*
Age, mean (±SD)	74.5 (10.8)	73.3 (12.2)	*0.107*
NIHSS at admission, median [IQR]	14 [8, 18]	13 [8, 18]	*0.101*
Multiple affected vascular territories, n (%)	1 (0.7)	1 (0.9)	*0.023*
Time to admission in min, median [IQR]	94.0 [58.8, 175.5]	106.0 [62.0, 210.0]	*0.005*
mRS pre-hospital, n (%)			*0.222*
0-1	135 (88.8)	101 (86.3)	
>1	17 (11.2)	16 (13.7)	

* CEM, Coarsened exact matching; GA, General anaesthesia; SD, standardised difference; SMD, standardised mean difference

Table II: Patient baseline characteristics of the complete PSM-matched data set

	GA	non-GA	*SMD*
n=568	284	284	
Sex male, n (%)	160 (56.3)	147 (51.8)	*0.092*
Age, mean (±SD)	70.8 (14.4)	71.1 (14.0)	*0.025*
NIHSS at admission, median [IQR]	14 [8, 19]	13 [7, 18]	*0.195*
Multiple affected vascular territories, n (%)	44 (15.5)	37 (13)	*0.071*
Time to admission in min, median [IQR]	140 [69.2, 277.5]	136.5 [65.0, 262.8]	*0.063*
mRS pre-hospital, n (%)			*0.136*
0-1	251 (84.8)	246 (86.6)	
> 1	43 (15.2)	38 (13.4)	

* PSM, Propensity score matching; GA, General anaesthesia; SD, standardised difference; SMD, standardised mean difference

Table III: Distribution of the affected vascular territories, unmatched

	GA	non-GA	*SMD*
n=1284	851	433	
ACA, n (%)			*0.147*
bilateral	15 (1.8)	2 (0.5)	
left	39 (4.6)	16 (3.7)	
right	35 (4.1)	24 (5.5)	
no	762 (89.5)	391 (90.3)	
MCA, n (%)			*0.188*
bilateral	19 (2.2)	4 (0.9)	
left	410 (48.2)	204 (47.1)	
right	375 (44.1)	213 (49.2)	
no	47 (5.5)	12 (2.8)	
PCA, n (%)			*0.195*
bilateral	29 (3.4)	4 (0.9)	
left	25 (2.9)	7 (1.6)	
right	21 (2.5)	11 (2.5)	
no	776 (91.2)	411 (94.9)	

* GA, General anaesthesia; SMD, standardised mean difference; ACA, anterior cerebral artery; MCA, medial cerebral artery; PCA, posterior cerebral artery

Table IV: Distribution of the endovascular treatment modality and rates of intravenous bridging thrombolysis, unmatched

	GA	non-GA	*SMD*
n=1284	851	433	
type of intra-arterial therapy, n (%)			
rtPA used	30 (3.5)	21 (4.8)	*0.065*
Urokinase used	32 (3.8)	49 (11.3)	*0.288*
mechanical treatment (including ‡)	776 (92.7)	384 (89.1)	*0.126*
bridging IVT, n (%)	519 (61.0)	234 (54.0)	*0.141*

* Patients may have received more than one intra-arterial treatment modality.

† GA, General anaesthesia; SMD, standardised mean difference; IVT, intravenous thrombolysis

‡ mechanical treatment including stent retriever, aspiration, distal retriever; with or without balloon angioplasty, permanent intracranial stenting, extracranial stenting

Supplemental figures:

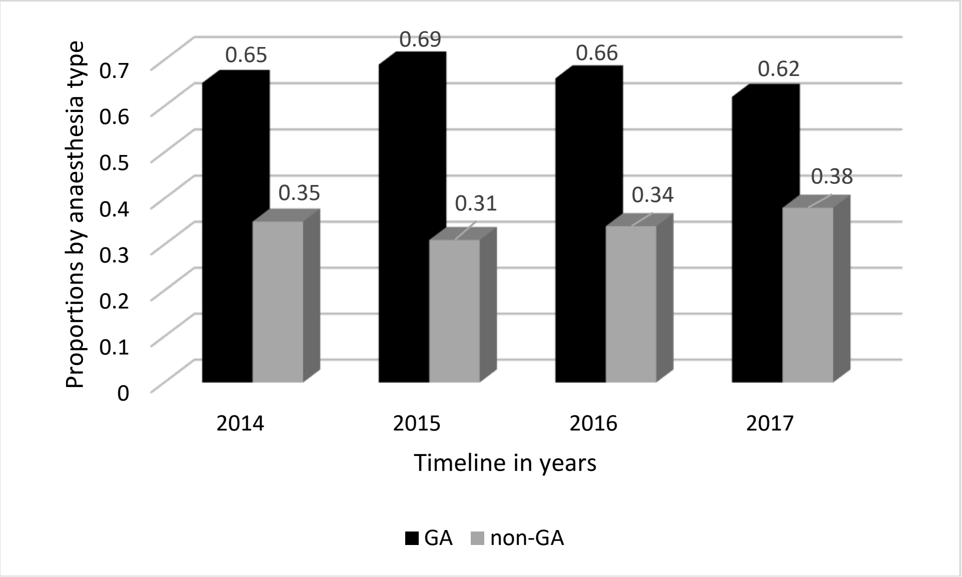

Figure I: Proportions of patients treated with and without GA from 2014 until 2017

* GA, General anaesthesia

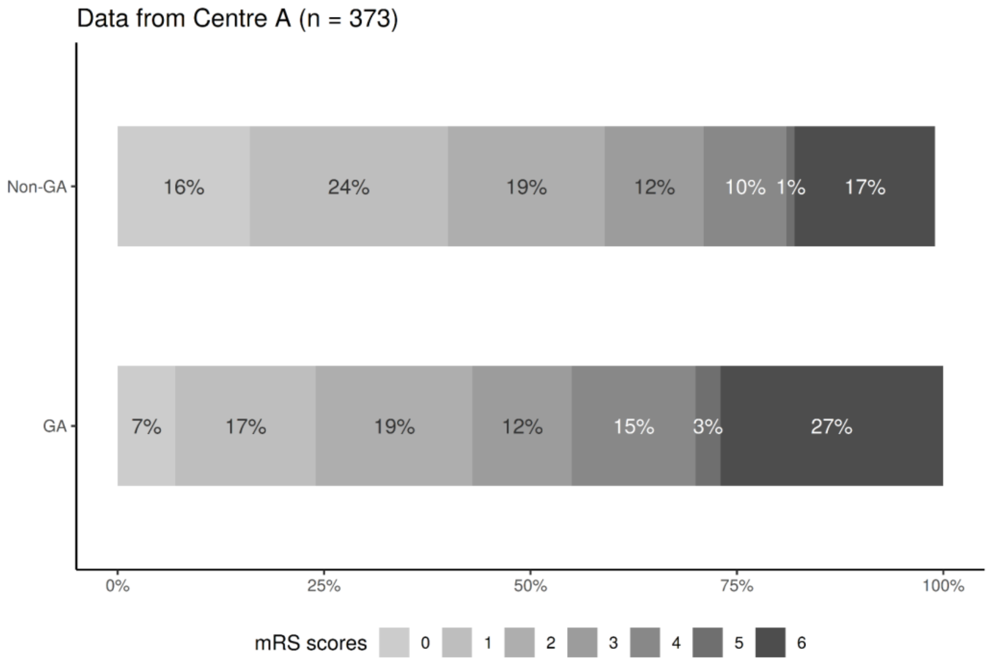

* Non-GA: n = 139; GA: n = 234

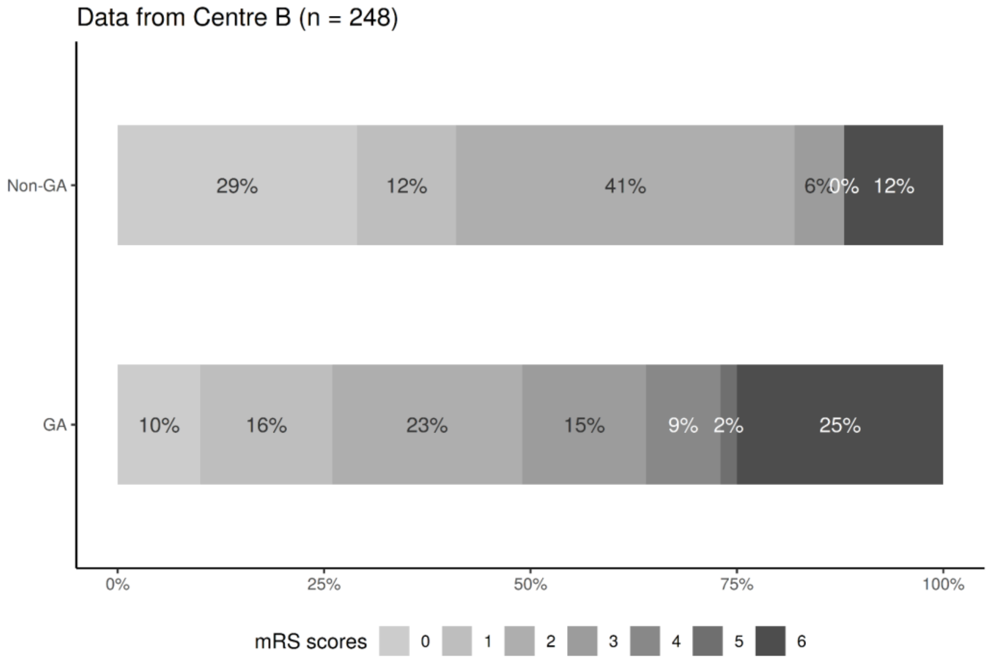

* Non-GA: n = 17; GA: n = 231

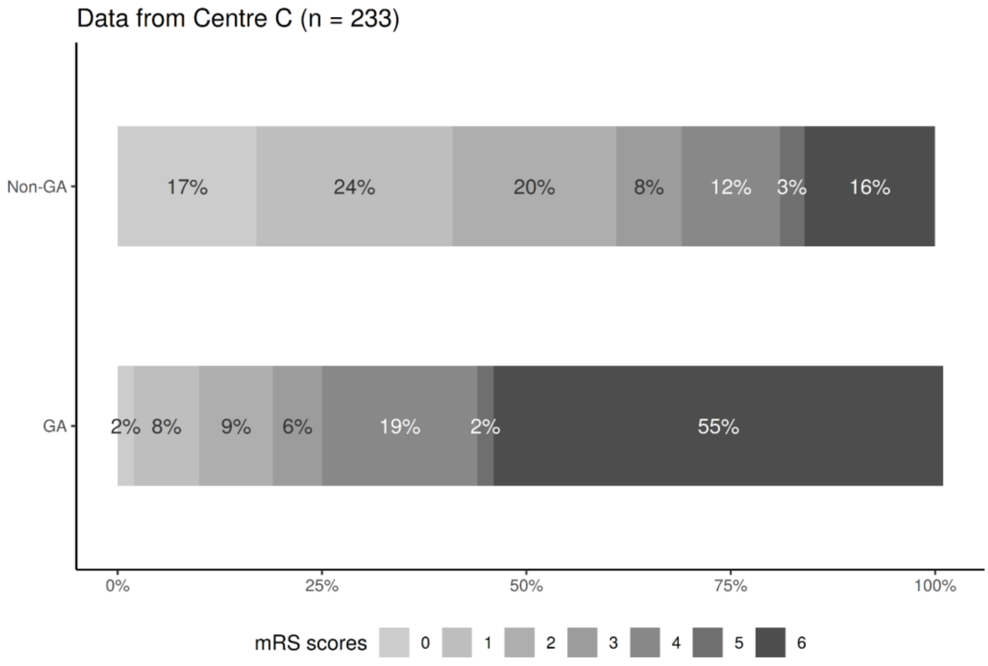

* Non-GA: n = 180; GA: n = 53

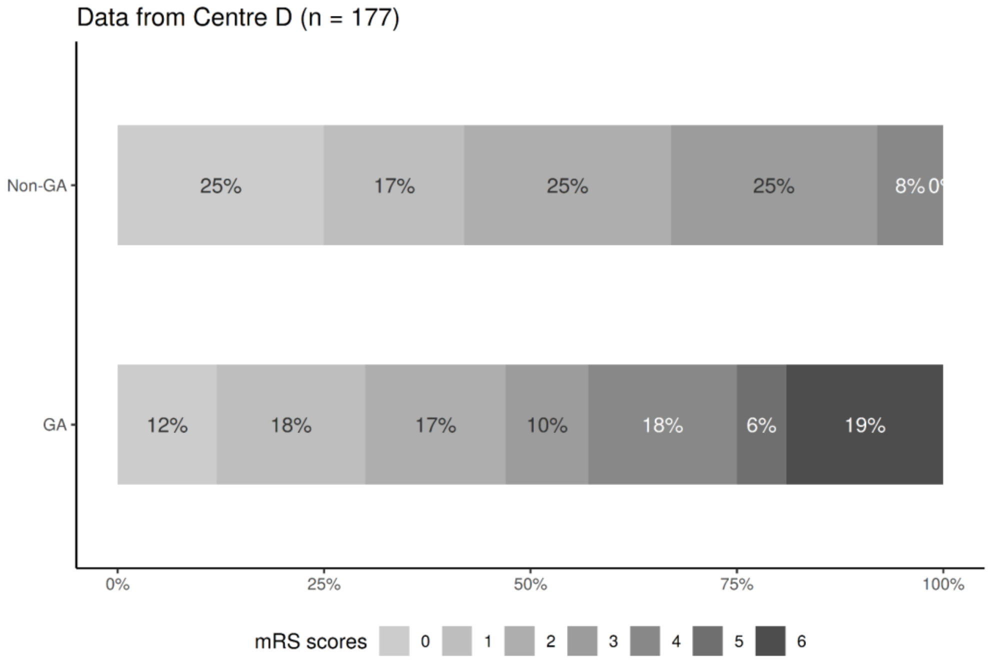

* Non-GA: n = 12; GA: n = 165

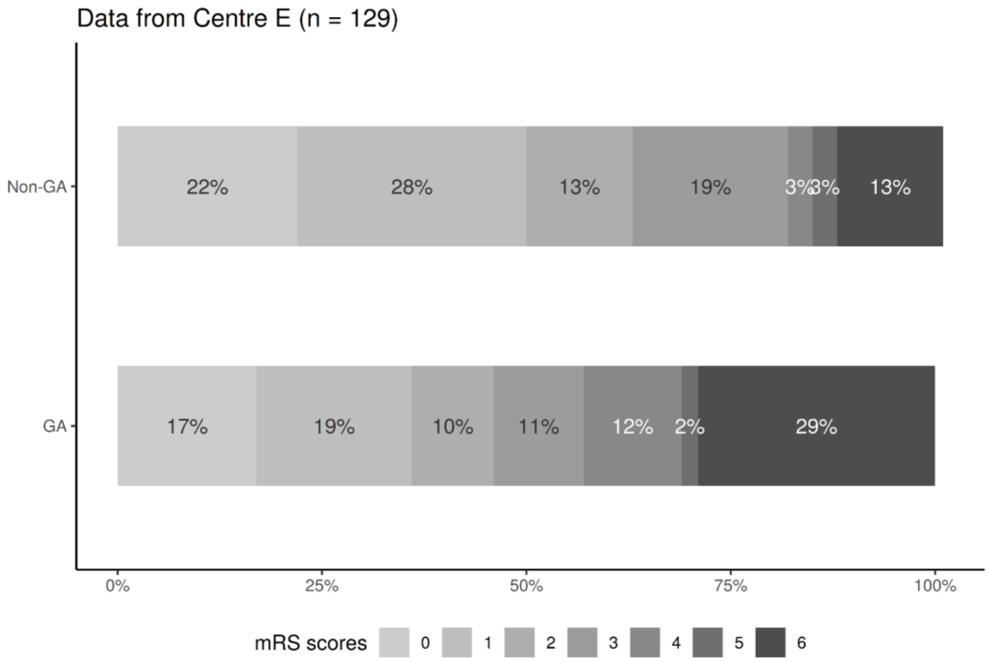

* Non-GA: n = 32; GA: n = 97

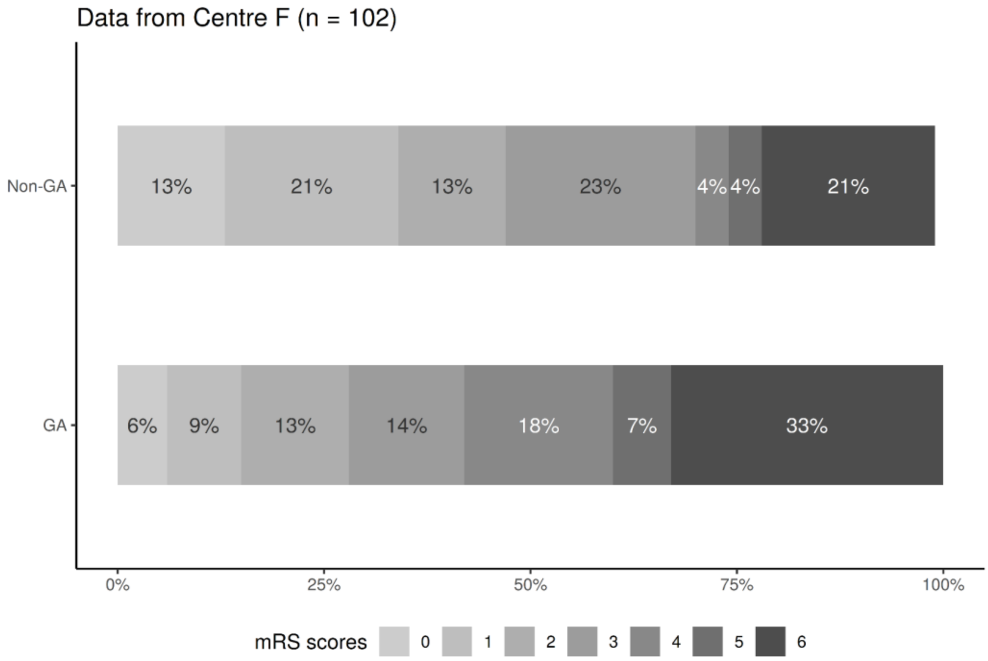

* Non-GA: n = 47; GA: n = 55

Figure II: Distribution of modified Rankin Scale (mRS) 3 months after stroke by type of anaesthesia in the 6 largest participating centres, unmatched

* GA, General anaesthesia

† mRS: 0= no symptoms, 1= no significant disability, 2= slight disability, 3= moderate disability, 4= moderately severe disability, 5= severe disability, 6= dead

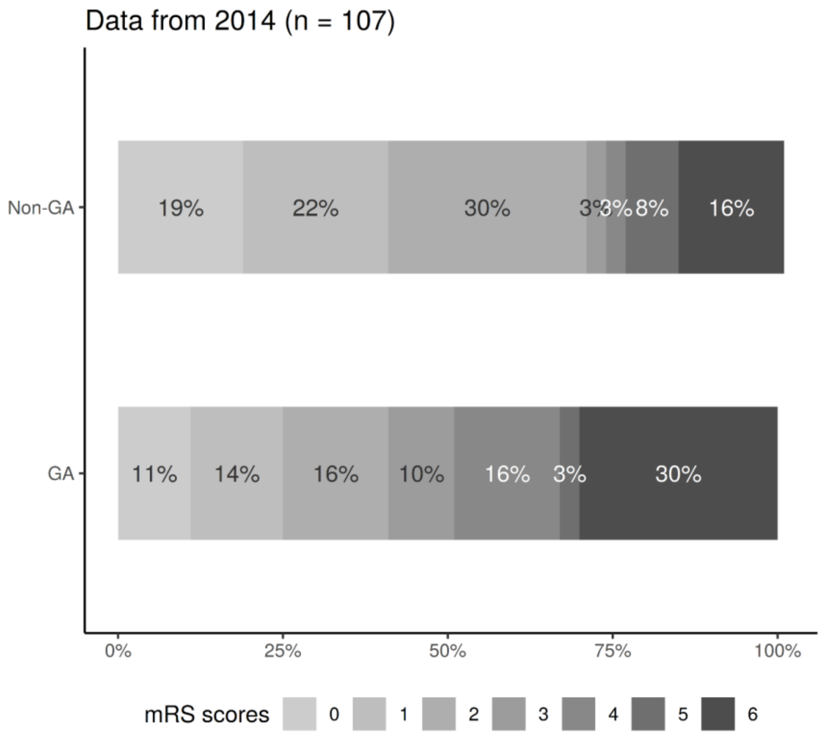

* Non-GA: n = 37; GA: n = 70

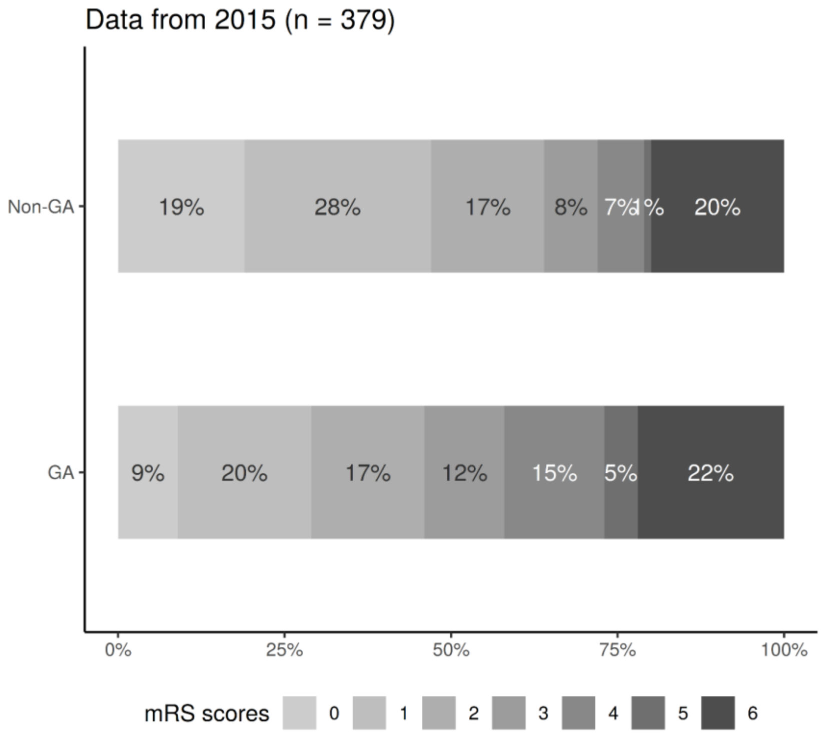

* Non-GA: n = 116; GA: n = 263

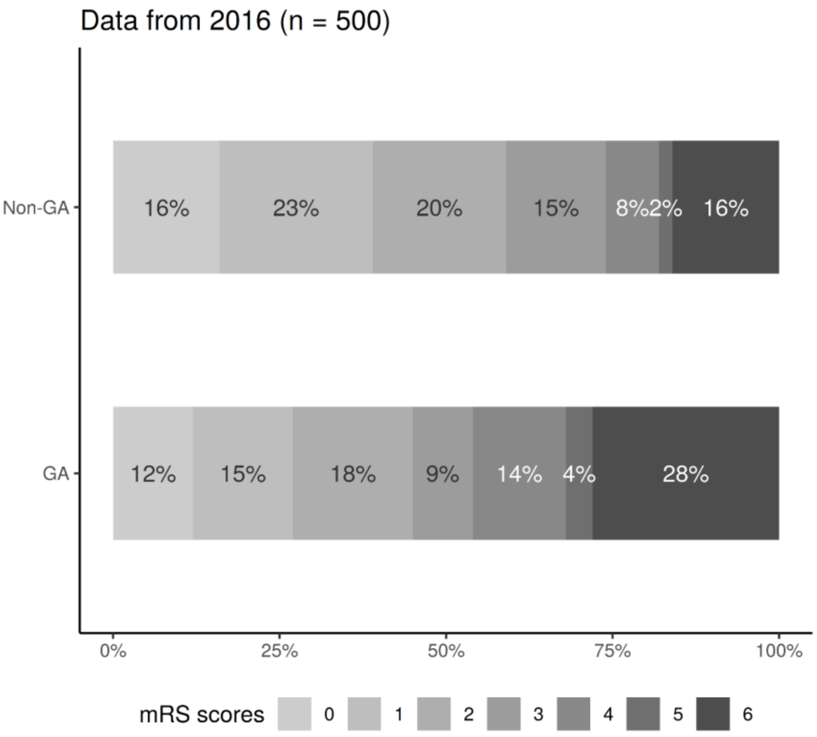

* Non-GA: n = 168; GA: n = 332

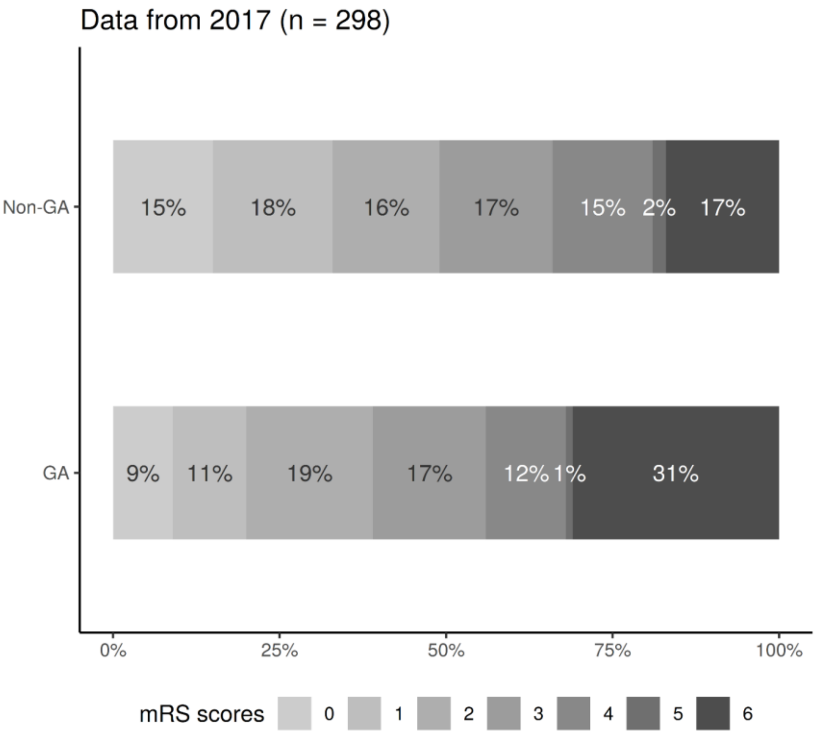

* Non-GA: n = 112; GA: n = 186

<u>Figure III:</u> Distribution of modified Rankin Scale (mRS) 3 months after stroke by type of anaesthesia from 2014 to 2017, unmatched

* GA, General anaesthesia

† mRS: 0= no symptoms, 1= no significant disability, 2= slight disability, 3= moderate disability, 4= moderately severe disability, 5= severe disability, 6= dead
